# Emotional quality-of-life after chest masculinizing surgery among transgender persons aged under versus over 18 years: a comparative observational study

**DOI:** 10.1186/s41687-026-01046-9

**Published:** 2026-03-23

**Authors:** Marine Bouron, Aurélie Bourmaud, Sehomi Azonaha, Elodie Fiot, Marie-Agathe Trouvin, Claire Vandendriessche, Anne-Sophie Lambert, Laetitia Martinerie

**Affiliations:** 1 Médecine de l’Adolescent, Hôpital Universitaire Bicêtre Paris Saclay, Assistance Publique - Hôpitaux de Paris, Paris, France; 2 Unité d’Épidémiologie Clinique, Hôpital Universitaire Robert-Debré, Assistance Publique - Hôpitaux de Paris, Paris, France; 3 UFR de Médecine, Université Paris Cité, Faculté de Santé, Paris, France; 4 Endocrinologie Pédiatrique, Centre de Référence Maladies Endocriniennes Rares de la Croissance et du Développement, Hôpital Universitaire Robert-Debré, Assistance Publique - Hôpitaux de Paris, Paris, France; 5 Plateforme Trajectoires Jeunes Trans, Paris, France; 6 Physiologie et Physiopathologie Endocriniennes, Inserm, Le Kremlin Bicêtre, France

**Keywords:** Adolescent, Gender identity, Quality-of-life, Sex reassignment surgery, Transgender

## Abstract

**Background:**

This study aimed to compare emotional quality-of-life among young transgender men who underwent chest masculinizing surgery before or after the age of 18, with a minimum postoperative follow-up of two years. Secondary objectives included comparison of surgical satisfaction and variation of quality-of-life depending on the number of years since the surgery.

**Methods:**

This cross-sectional, multicenter study was conducted in France between February 1st, 2024, and July 31st, 2024. Participants were aged 16 to 25 years and had undergone chest masculinizing surgery at least two years prior to inclusion. They were divided into two groups according to age at surgery: those operated before 18 years and those operated after 18 years. Participants completed an anonymous online questionnaire including items from the “Pediatric Quality of Life Inventory” and the “Trans-Questionnaire”. Emotional quality-of-life, satisfaction with surgery, and variation of emotional quality-of-life depending on the number of years since surgery were assessed. Between-group comparisons were performed using Wilcoxon tests for continuous variables and Fisher’s exact tests for categorical variables. A p-value < 0.05 was considered significant.

**Results:**

Forty-nine participants were included, with 22 individuals operated before the age 18 and 27 individuals operated after the age 18. The median follow-up period was 3.5 years. No significant difference in emotional quality-of-life was found between the groups (*p* = 0.47). There was no variation in quality-of-life scores according to time since surgery. Postoperative satisfaction was high: 100% of participants were satisfied with their chest appearance when dressed, 95% with their appearance when unclothed, 93% with scar location, and 91% with scar appearance.

## Introduction

In recent years, growing reflection on gender identity has been observed across Europe and around the world [1, 2]. In France, 2.3% of women and 2.4% of men in the adult population report having considered changing gender, a proportion that increases to 6% among individuals aged 18–29 years [3]. Specialized French clinics for the support of transgender minors have been established since 2012 [4]. Among transmasculine youth followed in these French pediatric clinics specializing in gender issues, approximately 20% request and subsequently undergo chest masculinizing surgery [4]. Epidemiological data on transgender adolescents in France are limited, although European and American studies report a substantial increase in the number of adolescents identifying as transgender and undergoing medical or surgical transition [1, 2]. Transgender adolescent boys report a significantly lower quality-of-life compared to their cisgender peers [5]. They are particularly at risk for suicide, with 50.8% reporting a history of suicide attempts [6]. Transgender adolescent boys are at higher risk of self-harm than transgender girls, with the risk increasing alongside gender dysphoria or body dissatisfaction, particularly related to breast development [7–9].

To reduce the distress associated with breast development, many individuals use chest binding techniques, involving mechanical compression to conceal their chest. But these techniques may lead to physical and functional complications [10–12]. Alongside these challenges, gender-affirming care, when provided in a comprehensive multidisciplinary setting (psychological, medical, and/or surgical), is associated with improvements in quality-of-life- [13, 14]. In its 2022 report, the World Professional Association for Transgender Health (WPATH) states that gender-affirming surgery may be considered for appropriately selected transgender adolescents when clinically and developmentally appropriate, following comprehensive multidisciplinary evaluation and individualized decision-making [13]. Chest masculinizing surgery is the most common gender-affirming surgery performed on transgender men [15, 16]. Previous studies have highlighted the psychosocial significance of breast-related distress and the potential positive impact of chest masculinizing surgery on the mental health of transmasculine adolescents and young adults [14, 17]. However, most existing data derive from adult cohorts or short-term follow-up periods, and robust long-term outcome data specifically in adolescents remain limited.

Most studies on postoperative quality-of-life have focused on adults and on the first 12 months after surgery, assessing the immediate impact of surgery on reducing gender dysphoria and improving mental health. These studies report significant improvements in body satisfaction and reductions in depression and anxiety [18]. A few studies have followed participants for up to two years after chest masculinizing surgery and demonstrated that improvements in quality-of-life, self-confidence, self-acceptance, and satisfaction with physical appearance were maintained beyond the first year [19, 20].

The primary objective of this study was to compare quality-of-life, particularly with the emotional dimension of the Pediatric Quality of Life Inventory (PedsQL), in individuals who underwent chest masculinizing surgery before the age of 18 versus in adulthood, with a delay of at least two years after the operation. The secondary objective was to examine differences in PedsQL emotional scores according to time elapsed since chest masculinizing surgery. The final objective was to compare postoperative satisfaction between the two groups using the Trans-Questionnaire (TRANS-Q).

## Materials and methods

### Study design

An email inviting participation and providing a link to the questionnaire was distributed by the medical secretariat for transgender men followed up at recruitment centers and who had undergone chest masculinizing surgery. Each participant received one initial email invitation and a single reminder. The questionnaire was available from February 2024 to July 2024. Data were collected anonymously using LimeSurvey, a secure online survey platform. Participants were free to withdraw from the questionnaire at any time without providing justification. Because the number of eligible participants was inherently constrained by strict inclusion criteria and real-world availability, no a priori sample size estimation was conducted, and all those concerned were invited to participate. The principal investigator remained blinded during the entire study period.

### Study population

Eligible participants were transgender men aged ≤ 25 years at the time of study participation who had undergone chest masculinizing surgery at least two years earlier and were followed at one of the two recruiting centers. As healing can continue throughout the first postoperative year, we chose a minimum interval of two years for inclusion in our study to ensure participants were beyond this initial postoperative period [21, 22]. In France, the first surgeries were performed around 2017, providing a maximum follow-up of eight years at the time of our study. Exclusion criteria included inability to complete the online questionnaire (due to a language barrier or lack of suitable equipment).

### Socio-demographic and surgical data

Socio-demographic data were collected through the questionnaire, including date of birth, age at questionnaire completion, date of surgery, age at surgery, and surgical indication (transgender identity, persistent gynecomastia, or other). These variables were used to calculate the time elapsed between surgery and questionnaire completion. Concerning the item on surgical indication, this approach aimed to avoid excluding individuals who did not specifically identify as transgender men but as other gender-diverse identities (e.g., gender-fluid, nonbinary, agender) who had also undergone chest masculinization surgery. In both recruiting centers, eligibility for chest masculinization surgery followed a multidisciplinary pathway consistent with the WPATH Standards of Care: prior hormone therapy was not a prerequisite for surgery, and decisions for adolescents were individualized through comprehensive evaluation and shared decision-making.

### Quality-of-life and post-surgery satisfaction

The PedsQL is a generic instrument designed to assess health-related quality-of-life across different populations, whether healthy or living with various conditions [23, 24]. There is no universal cut-off defining “good” or “poor” quality-of-life, as scores depend on multiple contextual factors. The PedsQL is most often used to compare groups within a given study, with the minimal clinically important difference (MCID) indicating the smallest change considered clinically meaningful [23, 25]. Quality-of-life was assessed using the French version of the PedsQL 4.0, adapted for individuals aged 18–25 years. The questionnaire includes four dimensions: physical functioning, emotional functioning, social functioning, and school/work functioning. Each item is rated on a 5-point Likert scale, from 0 (never a problem) to 4 (almost always a problem). Responses are then converted to a 0-100 scale, with higher scores reflecting better quality-of-life [23, 26, 27]. This questionnaire is recognized for its discriminative capacity, particularly for the emotional dimension [28]. For this dimension, the MCID has been estimated at 8.9 points [23]. The PedsQL scores were calculated according to the official scoring guidelines. Scale scores were computed when at least 50% of the items within the scale were completed. For the emotional dimension (5 items), a score was therefore calculated when at least three items were answered. When this threshold was met, the scale score was calculated as the mean of the completed items, as recommended in the PedsQL scoring manual [29]. The statistical analyses in this study focus specifically on this emotional dimension. The five items of the emotional functioning subscale are: “I feel afraid or scared”, “I feel sad or blue”, “I feel angry”, “I have trouble sleeping”, “I worry about what will happen to me” [27].

Postoperative satisfaction was assessed using the TRANS-Q, developed by Wanta et al. in 2019. This questionnaire is adapted from the BREAST-Q (which measures satisfaction in women after mastectomy) to better reflect the experiences of transgender men following chest surgery [30]. Each item is rated on a 5-point Likert scale (Strongly agree, Agree, Neutral, Disagree, Strongly disagree). From the TRANS-Q, six items related to postoperative satisfaction are selected, focusing on aesthetic and functional domains. Items were translated into French by the research team with minimal adaptations to ensure comprehension while preserving semantic equivalence. To enhance linguistic and cultural relevance, the translated version was also reviewed by representatives from transgender community associations to confirm the accuracy and appropriateness of the terminology used. The selected items are: “I am satisfied with location of my scars,” “I am satisfied with how my scars look”, “I am satisfied with how my nipples look”, “I am satisfied with how my chest looks with clothes on”, “I am satisfied with how my chest looks with clothes off”, “I feel comfortable/at ease during sexual activity” and “I would encourage others in a similar situation to seek surgery” [30]. In addition, participants were invited to provide open-ended comments at the end of the questionnaire.

### Statistical analyses

Participants were separated into two groups: the “under 18 years” group, including participants who underwent surgery before the age of 18 years, and the “over 18 years” group, including those who underwent surgery at the age of 18 or older. PedsQL emotional scores were compared between groups using Wilcoxon rank-sum tests. The variation of PedsQL emotional scores according to the time elapsed since surgery was analyzed using linear regression. Given the cross-sectional design, the results reflected between-participant differences in scores according to the number of years elapsed between surgery and questionnaire completion. Responses to the TRANS-Q questionnaire were analyzed separately for the two groups using Fisher’s exact test. All statistical tests were conducted using a significance threshold of *p* < 0.05. Statistical analyses were performed using RStudio software (version 2026.01.1–403).

### Ethics

This observational study was approved by the Local Ethics Review Committee for Biomedical Research Projects (No. 2023-645bis, IRB 00006477). All procedures were conducted in accordance with the Declaration of Helsinki and applicable national regulations. Informed consent was obtained from all participants. Although the minimum age for inclusion was 16 at the time of responding to the questionnaire, all respondents were at least 18 years old; therefore, parental or guardian consent was not required. This study is reported in accordance with the STROBE (Strengthening the Reporting of Observational Studies in Epidemiology) guidelines for cross-sectional studies [31].

## Results

**Fig. 1:**
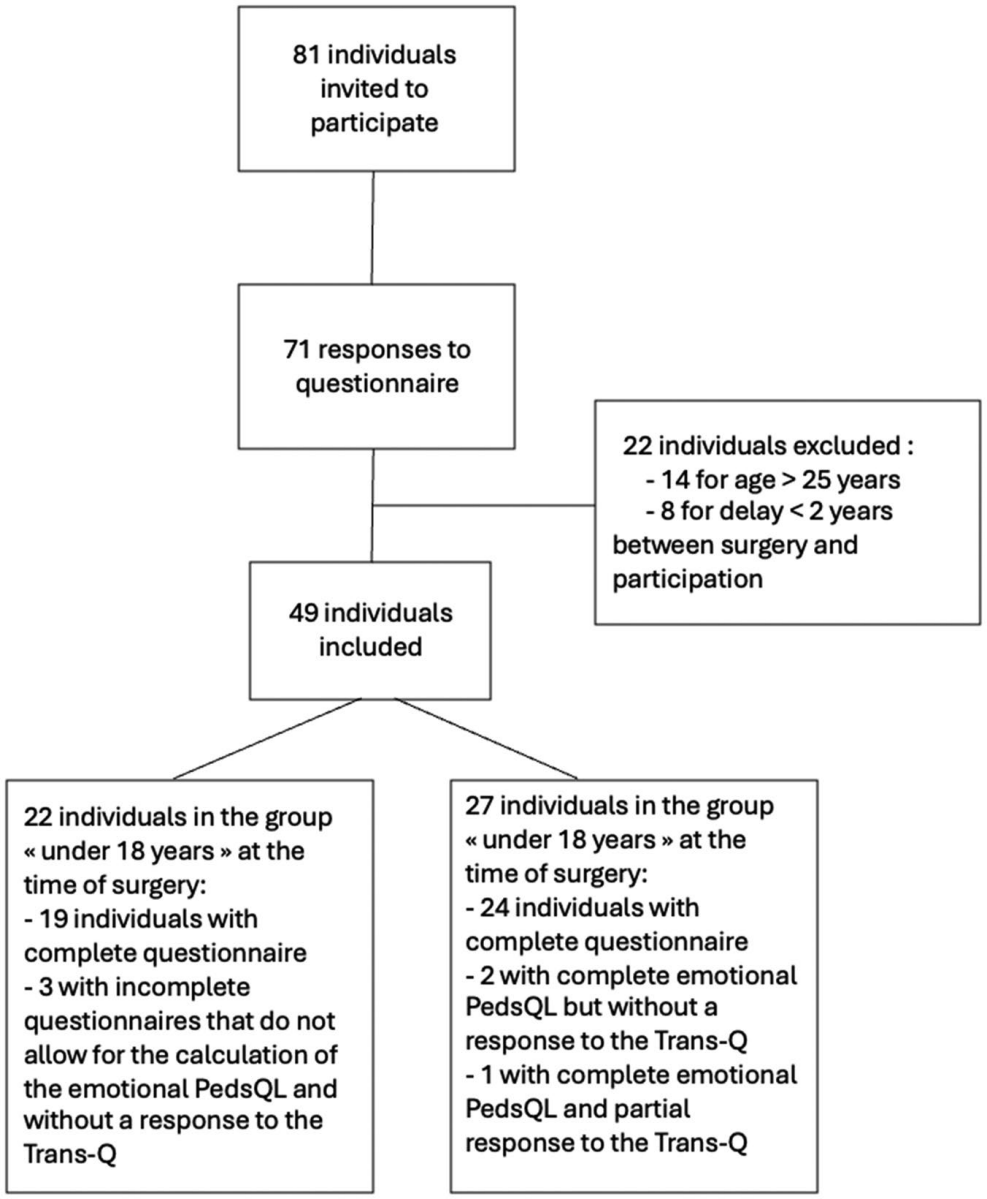
Flow-chart of study participants

**Fig. 2:**
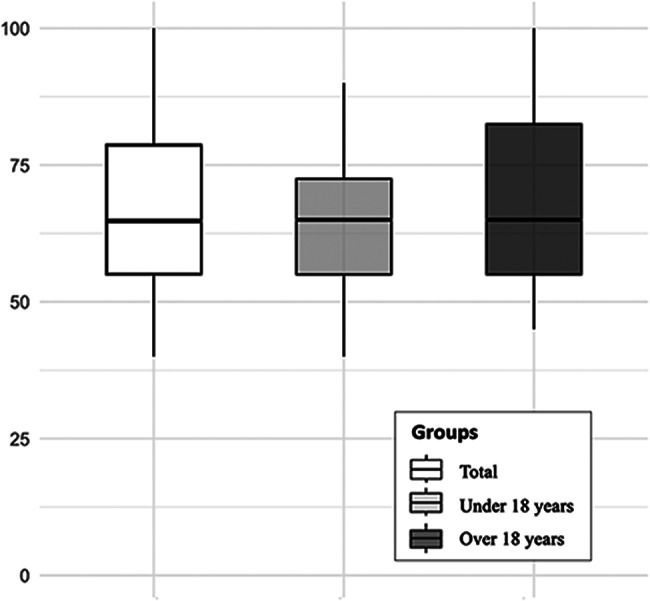
Results of emotional quality-of-life-scores (PedsQL)

The questionnaire was distributed to all individuals followed up at the recruiting centers who had had chest masculinizing surgery, representing 81 individuals. A total of 71 participants responded, representing a response rate of 88%. Among these respondents, those who did not meet the inclusion criteria regarding age or time since surgery were excluded. A total of 49 respondents were included in the study, with 22 in the “under 18 years” group at the time of surgery and 27 in the “over 18 years” group (Fig. 1). All participants reported undergoing chest masculinizing surgery for reasons related to transgender identity. The median age at inclusion was 21.4 years (interquartile range (IQR): 19.9–23.3) and the median age at surgery was 18.2 years (IQR: 16.8–19.2) (Table 1). In the “under 18 years” group, the median emotional quality-of-life-score was 65/100 (IQR: 55–73), and in the “over 18 years” group it was also 65/100 (IQR: 55–86). No significant difference was observed between the groups (*p* = 0.47) (Table 2; Fig. 2).

**Table 1:** Participant characteristics

Variables	Under 18 years *N* = 22	Over 18 years *N* = 27	Overall *N* = 49
**Age at surgery**			
Mean (SD) Median (IQR) Minimum-Maximum	16.5 (0.9) 16.5 (16.1–17.3) 14.5–17.7	19.3 (1.1) 19.2 (18.4–19.5) 18.0–21.5	18.0 (1.7) 18.2 (16.8–19.2) 14.5–21.5
**Age at questionnaire**			
Mean (SD) Median (IQR) Minimum-Maximum	20.1 (1.5) 19.8 (19.3–21.0) 18.0–23.2	23.1 (1.8) 23.3 (22.0–24.5) 20.3–25.7	21.8 (2.2) 21.4 (19.9–23.3) 18.0–25.7

**Table 2:** Results of emotional quality-of-life-scores (PedsQL)

	Under 18 years *N* = 19*	Over 18 years *N* = 27	Overall *N* = 46*	*p*-value^1^
**Emotional quality-of-life**				
Median (IQR)	65 (55–73)	65 (55–83)	65 (55–79)	0.47
Minimum-Maximum	40–90	45–100	40–100	

^1^Wilcoxon rank sum test between groups “under 18 years” and “over 18 years”

* The PedsQL score could not be calculated for three individuals in the “under 18 years” group due to a lack of responses from participants

**Fig. 3:**
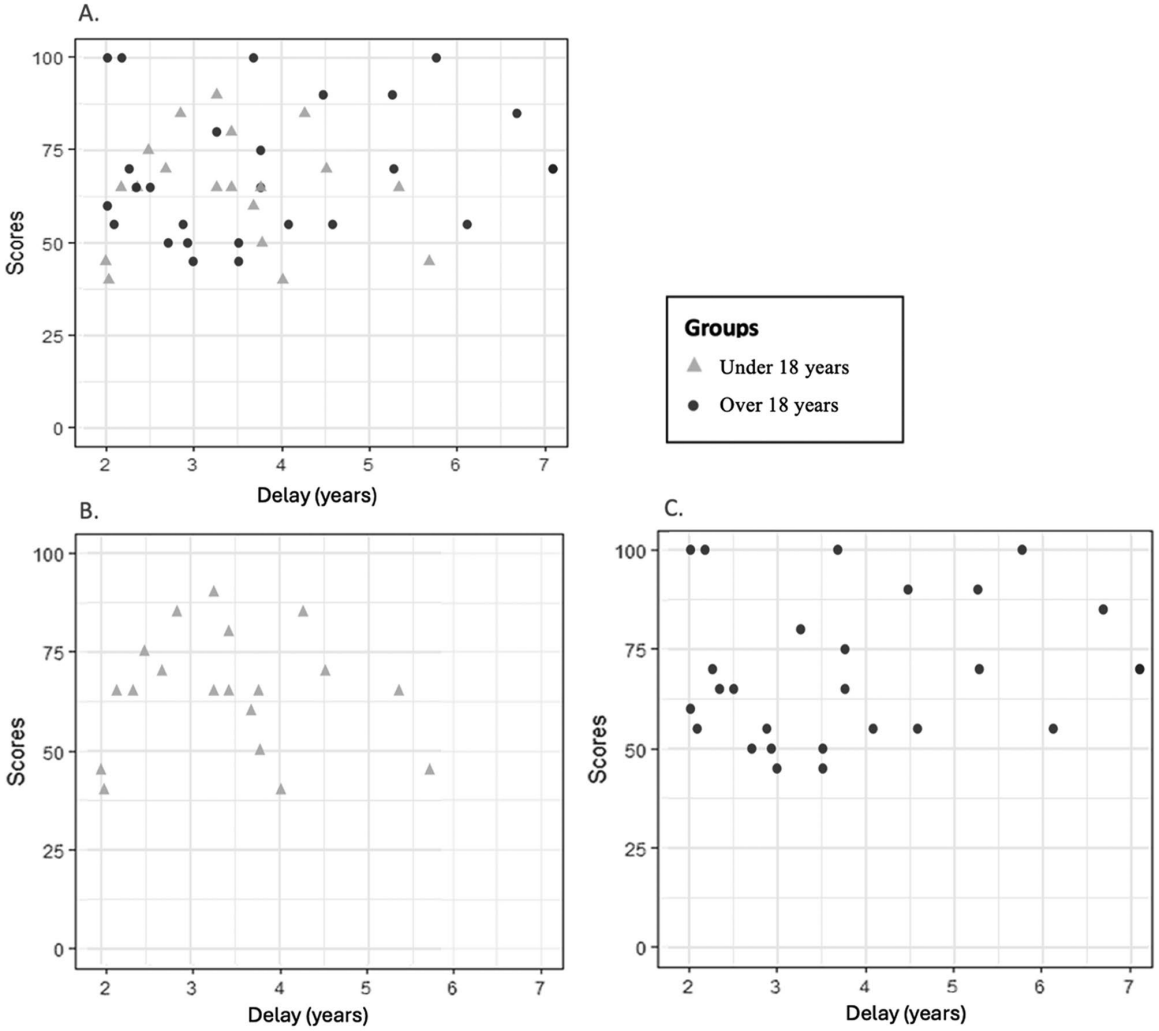
Emotional PedsQL scores by time elapsed since surgery. (**A**) In all participants; (**B**) In the under 18 years group; (**C**) In the over 18 years group

The number of years elapsed between surgery and questionnaire completion ranged up to 7.1 years, with an overall median of 3.5 years (IQR 2.5–4.5); more specially in group “under 18 years”: 3.4 [2.6–4.2] and in group “over 18 years”: 3.5 [2.6–4.9] (*p* = 0.61 between these two groups). Figure 3 presents a scatterplot showing emotional PedsQL scores as a function of time in years since surgery. No significant association was found between time since surgery and emotional PedsQL scores (β = 1.6; 95% CI: − 1.9 to 5.1; *p* = 0.37). Subgroup analyses also showed no significant association in the “under 18 years” group (β = − 0.48; 95% CI: − 7.3 to 6.3; *p* = 0.89) and in the “over 18 years” group (β = 1.9; 95% CI: − 2.4 to 6.3; *p* = 0.39).

Table 3 compares the results obtained by the “under 18 years” and “over 18 years” groups on the TRANS-Q items. Overall, 100% of participants reported satisfaction with chest appearance when dressed, and 95% with chest appearance when undressed (89% in “under 18 years” group versus 100% in the “over 18 years” group; *p* = 0.25). No participant disagreed with the statement “I would encourage others in a similar situation to seek surgery,” and none mentioned any feelings of regret in the open-ended comments at the end of the questionnaire (Appendix 1).

**Table 3:** Responses to TRANS-Q items

	Under 18 years	Over 18 years	Total	*p*.value^1^
	*N* = 19*	*N* = 25*	*N* = 44*	
**I am satisfied with location of my scars**				
Strongly agree / Agree	17 (89%)	24 (96%)	41 (93%)	
Neutral	1 (5%)	1 (4%)	2 (5%)	
Strongly disagree / Disagree	1 (5%)	0 (0%)	1 (2%)	0.90
**I am satisfied with how my scars look**				
Strongly agree / Agree	16 (84%)	24 (96%)	40 (91%)	
Neutral	1 (5%)	0 (0%)	1 (2%)	
Strongly disagree / Disagree	2 (11%)	1 (4%)	3 (7%)	0.76
**I am satisfied with how my nipples look**				
Strongly agree / Agree	16 (84%)	19 (76%)	35 (79%)	
Neutral	1 (5%)	2 (8%)	3 (7%)	
Strongly disagree / Disagree	2 (11%)	4 (16%)	6 (14%)	0.54
**I am satisfied with how my chest looks with clothes on**				
Strongly agree / Agree	19 (100%)	25 (100%)	44 (100%)	
Neutral	0 (0%)	0 (0%)	0 (0%)	
Strongly disagree / Disagree	0 (0%)	0 (0%)	0 (0%)	/
**I am satisfied with how my chest looks with clothes off †**				
Strongly agree / Agree	17 (89%)	24 (100%)	41 (95%)	
Neutral	2 (11%)	0 (0%)	2 (5%)	
Strongly disagree / Disagree	0 (0%)	0 (0%)	0 (0%)	0.25
**I am comfortable/at ease during sexual activity †**				
Strongly agree / Agree	15 (69%)	16 (67%)	29 (67%)	
Neutral	0 (0%)	2 (8%)	2 (5%)	
Strongly disagree / Disagree	1 (5%)	3 (12%)	4 (9%)	
Prefers not to answer	5 (26%)	3 (12%)	8 (19%)	0.34

^1^ Fisher’s exact test between groups “under 18 years” and “over 18 years”

* Five participants (three in the “under 18 years” group and two in the “under 18 years” group did not respond to any of the TRANS-Q

† One other participant in the “over 18 years” group did not respond to this item

The responses of individuals initially excluded because they were over 25 years of age at the time of the questionnaire are available for informational purposes in Appendices 2 and 3.

## Discussion

This study assessed the quality-of-life of transgender individuals who underwent chest masculinizing surgery before or after the age of 18 years. Emotional dimension scores did not differ significantly between the “under 18 years” and “over 18 years” groups (*p* = 0.47).

Whereas MCID has been estimated at 8.9 points for emotional quality-of-life-scores, in our study, the median emotional score was identical in both groups (65/100) [23]. As a reference, normative data from cisgender adolescents and young adults (18–25 years) report mean emotional functioning scores of approximately 67/100, a result that closely aligns with the scores observed in our study [32]. Concerning more specifically transgender adolescents, Zou et al. evaluated quality-of-life using the PedsQL in a center dedicated to transgender care, including individuals at various stages of transition (mean age: 15.7 years). The lowest scores were reported in the emotional dimension, with a median of 50/100 (IQR: 35–70) [5]. By comparison, the “under 18 years” group in our study had a higher median emotional score of 65/100. This score improvement may reflect greater societal support for transgender individuals in recent years, cultural differences between France and the USA, or the fact that our sample consisted exclusively of individuals who had undergone chest surgery - supporting the benefit of this surgery on the quality-of-life of transgender individuals. Transgender adolescents are particularly vulnerable to violence, marginalization, suicide attempts, and self-harming behaviors, often exacerbated by body dysphoria, especially related to breast development [33–36]. Given that existing evidence supports chest masculinizing surgery in transgender adults, and that our findings show no difference in outcomes when performed before or after age 18, there appears to be no justification for discouraging access to this surgery among adolescents who express the need for it.

In addition, our results show no significant association between PedsQL emotional scores and time in years since surgery. This cross-sectional finding indicates that participants assessed at different postoperative durations reported no different levels of emotional quality-of-life. After medical or surgical treatments of major personal significance, some studies suggest the phenomenon of hedonic adaptation, whereby quality-of-life initially improves, but individuals gradually readjust their internal standards, leading to a subsequent decline in perceived quality-of-life [37–39]. In our study, emotional quality-of-life scores did not decline with increasing time since surgery, which does not support the occurrence of hedonic adaptation in this context and suggests that the benefits of chest masculinizing surgery are sustained over time. A related and more extensively operationalized concept in patient-reported outcomes research is response shift, defined as a change in the meaning of one’s self-evaluation of a target construct resulting from recalibration of internal standards, reprioritization of values, or reconceptualization of the construct itself, following a significant health event [40–42]. In the context of chest masculinizing surgery — a personally transformative intervention — participants may progressively reassess their expectations and redefine their reference points for quality-of-life over time. However, as response shift is inherently a time-dependent, within-person phenomenon, it cannot be formally assessed in a cross-sectional design. Future longitudinal studies would be valuable to more precisely characterize individual trajectories.

All participants reported being fully satisfied with the appearance of their chest when dressed. In previous studies, 85% of transgender adolescents who had not undergone chest masculinizing surgery reported fear of having their chest seen, and 74% avoided bathing in public places [14, 43]. A majority expressed the feeling that life had not yet begun due to breast development, and many report that their chest dysphoria causes strong negative emotions that can be coupled with suicidal thoughts [14, 36, 44]. The literature also highlights that transgender individuals often feel unsafe in public spaces, particularly because of the appearance of their chest [14, 43]. In our study, 95% of participants reported satisfaction with their chest appearance when undressed. This finding is consistent with previous studies reporting an association between chest masculinizing surgery and higher body image satisfaction [10, 36]. A total of 67% of participants in our study reported feeling comfortable during sexual activity. Discomfort during sexual activity may also be influenced by other dimensions of gender dysphoria, such as genital dysphoria, as well as by factors not assessed in the present study, and therefore may not be fully explained by chest-related satisfaction alone. Conversely, in a population that had not undergone chest masculinizing surgery, more than half experienced difficulties in their intimate relations [14].

According to the WPATH Standards of Care, pelvic gender-affirming surgeries are not recommended before the age of 18 years, and chest masculinization surgery is the only gender-affirming surgical procedure that may be considered for adolescents under 18 years, following multidisciplinary evaluation and individualized decision-making [13]. Beyond accessibility constraints, transgender adolescents generally express a stronger desire for chest masculinization than for pelvic surgery when considering their future surgical transition [45]. It may be due to the greater social visibility of breast development [45]. To alleviate distress related to breast development, many transgender individuals use mechanical methods to conceal their chest (elastic bandages, tight sports bras or binders). Chest dysphoria can be so severe that up to 15% of young transgender people wear binders at night [14]. In the study by Mehringer et al., all participants reported using compression techniques prior to chest surgery. Although binder use may alleviate psychological discomfort, all subjects reported physical side effects [10]. These compression techniques caused pain in 74% of cases: mainly thoracolumbar, but also abdominal and shoulder pain. Additional side effects included skin damage (76%), poor posture (40%), shortness of breath (40%), lung infections (3%), and rib fractures (3%) [11]. Providing access to surgery for transgender boys could reduce the need for these compression techniques [11, 14]. Recent prospective data show that gender-affirming chest reconstruction significantly improves gender and appearance congruence and reduces chest dysphoria in adolescents and young adults [17]. This finding reinforces our results and underscores the importance of surgical access for this population.

During adolescence, cisgender boys may develop gynecomastia [46, 47]. When gynecomastia persists and leads to psychosocial distress, surgical intervention may be indicated to restore a masculine chest shape. This surgery, which requires an individualized approach, is associated with a high rate of postoperative satisfaction, improved quality-of-life, and reduced emotional distress and interpersonal difficulties related to chest appearance [48–50]. In these situations, chest surgery is considered justified because the individual’s physical appearance does not align with his gender identity. In these situations, chest surgery is considered medically and ethically justified because the individual’s physical appearance causes significant psychological distress and does not align with his gendered self-perception [48–50]. The needs expressed by transgender adolescents are based on the same principle: in both cases, the goal is to alleviate distress related to incongruence between body and gendered self-image, and to support psychological well-being and social functioning [51]. According to the principle of non-discrimination in medical ethics, individuals should not receive different treatment based on personal characteristics such as origin, family situation, or gender identity [51, 52]. Denying access to chest surgery for transgender adolescents—while offering surgery to cisgender peers—may therefore constitute discrimination based on gender identity [51]. Although our questionnaire included the option to indicate gynecomastia surgery (none of the participants selected this option), future studies could administer the same instrument to cisgender young men operated on for gynecomastia to directly compare postoperative quality-of-life and surgery satisfaction outcomes with transgender peers.

No participant disagreed with the item " I would encourage others in a similar situation to seek surgery”. In the open comments at the end of our questionnaire, none of the participants mentioned any regrets or the realization of reversal surgery, although regret was not assessed directly. Satisfaction and regret are related but distinct concepts, with high satisfaction and minimal regret typically reported in adolescent gender-affirming care [53]. A systematic review estimated an overall regret rate of 0.8% among transmasculine individuals across all types of gender-affirming procedures [54]. Another systematic review reported a regret rate of < 1% focused on chest masculinizing surgery [55].

### Limitations

One limitation of our study is the small sample size. Indeed, a posteriori statistical power was calculated and estimated at 20%, which is low. This small sample size reflects the rarity and recent nature of the population concerned, for which we sought all eligible subjects. The study should therefore be interpreted as exploratory and descriptive, providing preliminary results that will require confirmation in larger cohorts or multicenter studies. As a result, our study was unable to demonstrate any difference between our two groups, if one existed. However, despite this limitation, the absolute figures identified in these small samples do not reflect a clinically relevant trend towards difference. While the sample size is relatively small, the cohort provides extended postoperative follow-up in adolescents, a population for which longitudinal outcome data remain limited. As such, these findings contribute meaningfully to a field in which long-term evidence is still emerging, and lay the groundwork for larger prospective studies.

Other limitations concern the use of a non-validated French translation of the TRANS-Q and the absence of detailed information on the surgical techniques used. The anonymous survey design prevented collection of such data or linkage with medical records, although including this information would have provided valuable insight into potential variations in postoperative experiences.

This study may also be subject to selection bias, as participation was voluntary and potentially more appealing to individuals satisfied with their surgical outcomes or who felt comfortable discussing their experience. Some individuals might have emphasized positive outcomes to avoid limiting access to surgery for others. Nevertheless, the anonymous survey design and absence of direct investigator contact likely encouraged broad participation, and variability observed in PedsQL scores suggests the inclusion of diverse experiences. Gender identity was assessed only at the time of surgery indication and not longitudinally; therefore, potential evolution of gender identity over time could not be examined. Moreover, gender identity was assessed using the item on surgical indication (transgender identity, persistent gynecomastia or other). Although this question was intended to distinguish transgender male identities from cisgender male identities, participants identifying as non-binary, genderqueer, or gender-fluid may have selected ‘transgender’ as the most accessible option, despite the possibility of responding ‘other.’ As a result, the diversity of gender identities may have been underrepresented. Furthermore, the intentional omission of detailed sociodemographic variables, including socioeconomic status, education level, and employment status, limits the ability to fully characterize the study population and assess the generalizability of the results to broader clinical settings. This decision was made in order to preserve the anonymity of participants, given the small size and potentially identifiable nature of the clinical population, and to minimize the burden on respondents on a sensitive topic. However, in future studies with larger sample sizes, it would be interesting to collect additional demographic data.

## Conclusion

This study did not identify significant differences in quality-of-life between transgender individuals who underwent chest masculinizing surgery before or after the age of 18, and the scores did not vary significantly according to the number of years since surgery. Moreover, postoperative satisfaction was high in both groups, with no statistically significant difference observed. These results were observed among transgender individuals who expressed the need for surgery and were followed within multidisciplinary teams experienced in transgender care. As the transgender youth population is particularly at risk of suicide, self-harm, and violence, allowing them to access this surgery, according to their needs without necessarily waiting until adulthood, could be beneficial to their mental health. As gender identity is deeply personal, individualized and patient-centered care remains essential.

**Appendix 1:** Free-Text Comments of participants

Comment in French (original language)	Comment translated into English	Age of participant at time of surgery
Je ne pense pas pouvoir “recommander” à qui que ce soit une opération chirurgicale lourde, mais je peux dire que pour moi et selon mon expérience, c’était la meilleure chose à faire et je n’ai aucun regret.	I don’t think I can “recommend” major surgery to anyone, but I can say that for me and based on my experience, it was the best thing to do and I have no regrets.	18.7 years old
L’opération m’a beaucoup aidé dans mon rapport à mon corps, je suis beaucoup plus heureux et j’ai pris confiance en moi.	The operation has greatly assisted me in my relationship with my body; I am much happier and have gained confidence in myself.	17.5 years old
J’ai eu la chance d’avoir une chirurgie avec le moins de cicatrice. Clairement, si j’avais eu une chirurgie avec deux énormes cicatrices sur le torse, je ne pense pas que je l’aurais aussi bien vécu. Pour l’aspect des tétons, le soucis est que je vois le soucis moi-même mais je pense que de l’extérieur ça va. Je pense que pour le coup bien loin de tout, c’est une insatisfaction très bénigne.	I was fortunate to have surgery with minimal scarring. Clearly, if I had had surgery with two huge scars on my chest, I don’t think I would have coped so well. As for the appearance of my nipples, the problem is that I see the problem myself, but I think that from the outside it’s fine. I think that, all things considered, it’s a very minor dissatisfaction.	19.4 years old
La mammectomie a été une vraie délivrance et je suis reconnaissant d’avoir pu la faire aussi jeune (18 ans). J’ai aussi eu la chance d’avoir des parents qui pouvaient payer l’opération ce qui m’a permit de passer dans le privé et d’avoir beaucoup moins d’attente que dans le public (surtout qu’à cette époque c’était le covid). Je suis satisfait de tout mon parcours de transition dans la globalité et la mammectomie m’a vraiment permis de me sentir mieux avec mon corps. J’ai eu la péri aréolaire, j’ai donc seulement des cicatrices autours de mes tétons qui ne se voient quasiment pas et cela joue aussi beaucoup, j’aurais eu plus de mal avec de grosses cicatrices qui m’auraient rappeler chaque jour que j’avais une poitrine avant. Alors qu’à l’heure actuel j’oublie même que c’était le cas.	The mastectomy was a real relief, and I am grateful that I was able to have it done at such a young age (18). I was also lucky to have parents who could pay for the operation, which allowed me to go private and have a much shorter wait than in the public system (especially since it was during COVID at the time). I am satisfied with my entire transition journey overall, and the mastectomy really helped me feel better about my body. I had periareolar surgery, so I only have scars around my nipples, which are almost invisible, and that makes a big difference. I would have had a harder time with large scars that would have reminded me every day that I used to have breasts. Now, I even forget that I ever did.	18.2 years old
Je suis né le jour de cette opération. Je vis et j’ai arrêté de survivre.	I was born on the day of this operation. I am living, and I have stopped merely surviving.	16.9 years old
J‘apprécie beaucoup l’aide et le support que j’ai reçu de la part de ma famille et des médecins pendant ma transition.	I really appreciate the help and support I received from my family and doctors during my transition.	16.2 years old
J’ai été heureux d’avoir eu l’occasion de faire la mastectomie pendant mon adolescence (16 ans) !	I was happy to have had the opportunity to have a mastectomy during my teenage years (at age 16)!	16.0 years old
La torsoplastie a été comme une libération, je l’ai faite quelques mois après le commencement du traitement hormonal (testostérone), c’est à ce moment que j’ai vraiment senti une avancée dans ma transition, le fait de me regarder dans le miroir et de m’apprécier, de faire plus attention à moi et avoir plus confiance en moi. À partir de ça le regard des autres aussi	The torsoplasty was like a liberation. I had it done a few months after starting hormone treatment (testosterone), and that’s when I really felt a breakthrough in my transition, looking at myself in the mirror and liking what I saw, taking better care of myself and feeling more confident. From then on, other people’s perceptions changed too.	16.1 years old
C’était la meilleure opération de ma vie	It was the best operation of my life.	20.7 years old
Je suis atteint d’un trouble anxieux qui me provoque des difficultés au quotidien et explique certaines de mes reponses. Mais la torsoplastie ne m’a apporté que du positif et du mieux-être, et je suis beaucoup plus à l’aise dans mon corps depuis.	I suffer from an anxiety disorder that causes me difficulties in my daily life and explains some of my responses. But torsoplasty has brought me nothing but positive results and improved well-being, and I feel much more comfortable in my own body since the procedure.	20.7 years old
Très gros volume de poitrine de base, binder inefficace et douloureux à porter.	Very large natural breast size, ineffective binder that is painful to wear.	21.1 years olds

**Appendix 2:** Responses to TRANS-Q items for individuals who were over 25 years old at the time of the study (*n* = 11 participants with complete questionnaires)

**I am satisfied with location of my scars**	
Strongly agree / Agree	9 (82%)
Neutral	1 (9%)
Strongly disagree / Disagree	1 (9%)
**I am satisfied with how my scars look**	
Strongly agree / Agree	8 (73%)
Neutral	1 (9%)
Strongly disagree / Disagree	2 (18%)
**I am satisfied with how my nipples look**	
Strongly agree / Agree	7 (64%)
Neutral	2 (18%)
Strongly disagree / Disagree	2 (18%)
**I am satisfied with how my chest looks with clothes on**	
Strongly agree / Agree	11 (100%)
Neutral	0 (0%)
Strongly disagree / Disagree	0 (0%)
**I am satisfied with how my chest looks with clothes off**	
Strongly agree / Agree	9 (82%)
Neutral	1 (9%)
Strongly disagree / Disagree	1 (9%)
**I am comfortable/at ease during sexual activity**	
Strongly agree / Agree	7 (64%)
Neutral	0 (0%)
Strongly disagree / Disagree	3 (27%)
Prefers not to answer	1 (9%)

**Appendix 3:** Results of emotional quality-of-life scores (PedsQL) for individuals who were over 25 years old at the time of the study (*n* = 11 participants with complete questionnaires)

Emotional quality-of-life	
Median (IQR)	75 (58–90)
Minimum-Maximum	50–95

Nota Bene: It is important to note that the PedsQL questionnaire was originally developed and validated for individuals under the age of 25. Therefore, the results from participants over the age of 25 should be interpreted as exploratory and informative rather than validated results

## Data Availability

The datasets generated and analyzed during the current study are not publicly available due to confidentiality agreements with participants but are available from the corresponding author upon reasonable request. Due to copyright restrictions concerning PedsQL, the full questionnaire cannot be reproduced. Interested readers may obtain the official version from Mapi Research Trust (https://eprovide.mapi-trust.org).
